# Firearm Experiences, Behaviors, and Norms Among Rural Adolescents

**DOI:** 10.1001/jamanetworkopen.2024.41203

**Published:** 2024-10-24

**Authors:** Elizabeth H. Weybright, Heather F. Terral, Ashley Hall, Gary Varrella, Alice M. Ellyson, Julia P. Schleimer, Margaret R. Kuklinski, Kimberly Dalve, Emma L. Gause, Sabrina Oesterle, Ali Rowhani-Rahbar

**Affiliations:** 1 Department of Human Development, Washington State University, Pullman; 2 Firearm Injury & Policy Research Program, University of Washington, Seattle; 3 Department of Community and Behavioral Health, Elson S. Floyd College of Medicine, Washington State University, Pullman; 4 4-H Youth Development, Snohomish County Extension, Washington State University, Snohomish; 5 4-H Youth Development, Spokane County Extension, Washington State University, Spokane; 6 Department of Pediatrics, University of Washington, Seattle; 7 Center for Child Health, Behavior and Development, Seattle Children’s Research Institute, Seattle, Washington; 8 Department of Epidemiology, University of Washington, Seattle; 9 Social Development Research Group, School of Social Work, University of Washington, Seattle; 10 School of Social Work, Arizona State University, Phoenix

## Abstract

**Importance:**

Rural adolescents are at high risk for firearm-related injury, yet most existing prevention efforts are informed by research conducted in urban areas. Despite the need to account for rural perspectives, few studies have investigated the unique social ecological context of firearms for rural adolescents or have directly engaged with rural adolescents to understand their views on firearm use.

**Objective:**

To describe rural adolescents’ firearm behaviors and perceptions of firearm-related social norms within their communities, peer groups, and families.

**Design, Setting, and Participants:**

This cross-sectional study used a convergent mixed-methods design and involved a community-based participatory research approach. Participants comprised 93 adolescents residing in rural Washington state and enrolled in a county or tribal reservation 4-H youth development program as an intermediate or senior age-level grouping (ie, aged 12-19 years). Adolescents completed a survey and participated in a semistructured focus group or interview between September 1, 2021, and September 30, 2022. Data were analyzed using descriptive and thematic analysis.

**Main Outcomes and Measures:**

Individual handgun behavior and peer, family, and community perceptions of firearm-related social norms.

**Results:**

The sample included 93 adolescents (mean [SD] age, 15.7 [1.7] years; 49 female participants [52.7%]). Approximately half of participants (52 [55.9%]) had carried a handgun at some point in their lives, with a mean (SD) age at first carry of 10.9 (3.1) years. Primary themes across quantitative and qualitative data focused on social norms (specifically, acceptable and unacceptable ways to engage with firearms, reasons for carrying firearms, and places to carry a firearm).

**Conclusions and Relevance:**

In this cross-sectional study, rural adolescents in 4-H programs generally understood acceptable and unacceptable firearm carrying behaviors, which aligned with state laws. Findings provide context for rural adolescent behaviors such as carrying a firearm predominantly for prosocial reasons (hunting, recreation, and sport) and adult behavior such as carrying for protection of person and personal property. Understanding firearm-related social norms in rural communities holds implications for firearm injury prevention efforts, especially related to enhancing training, developing norms-based prevention approaches, and tailoring efforts to rural settings.

## Key Points

**Question:**

What are rural adolescents’ experiences with firearms and perceptions of firearm-related social norms?

**Findings:**

In this cross-sectional study of 93 adolescents in rural Washington state, 52 adolescents (55.9%) reported having ever carried a handgun, and they first carried a handgun at a mean age of 10.9 years. Adolescents identified social norms across community, peer, and family groups, which differentiated between acceptable and unacceptable handgun carrying behaviors, reasons for carrying, and places to carry a handgun.

**Meaning:**

Findings suggest adolescent firearm behaviors and social norms are associated with the rural context and would benefit from tailored, norms-based firearm injury prevention approaches.

## Teaser

This cross-sectional study describes rural adolescents’ experiences with firearms and perceptions of firearm-related social norms within their communities, peer groups, and families.

## Introduction

Rural adolescents are at greater risk than their urban counterparts for firearm-involved unintentional injury or death and firearm suicide.^1,2^ Research on firearms in rural communities finds that, compared with urban adolescents, rural adolescents have greater access to firearms, have higher rates of carrying firearms, and experience unique firearm-related contextual factors.^3,4,5^ For example, rural residences are more likely than urban residences to have firearms in the home and to store them unlocked and loaded.^6,7,8^ Given the risk of death by suicide and homicide associated with household access to firearms^9^ and limited access to health care in rural areas,^10^ firearm injury prevention strategies that are developmentally appropriate and effective for rural adolescents are greatly needed.

Grounded in the social ecological model^11,12^ and theory of planned behavior,^13^ we posit that rural adolescents encounter socializing experiences among their communities, families, and peers that are associated with their operationalization of firearm-related social norms, and ultimately, intentions and behaviors. These norms likely operate differently within rural communities than in urban areas. Social norms serve as unwritten, culturally bound rules influencing behaviors based on personal beliefs of others’ approval or disapproval of behaviors, such as carrying firearms (injunctive norms), or beliefs about how common a behavior is within their environment (descriptive norms).^14,15^ When an adolescent perceives a behavior as more acceptable and common, they are more likely to engage in that behavior. A prior study of adolescent firearm norms found differences among rural adolescents who reported having carried a handgun and those who did not, such that those who carried a handgun reported more approving injunctive norms (eg, “carrying a handgun is cool”) and descriptive norms (eg, number of friends who carried a handgun).^16^

Reliable insight into firearm-related norms is needed to develop prevention programs to address the needs and contexts of rural communities.^3^ Understanding adolescents’ firearm-related social norms precipitates understanding their firearm-related behaviors, including carrying firearms generally and to school. However, our understanding of these topics in rural areas is limited. Work investigating the acceptability of health care professionals asking patients about firearm access indicated that about two-thirds of patients found such screening at least sometimes appropriate.^17,18^ Acceptability of firearm-related conversations likely differs by who is asking, what is being asked, and cultural norms.^19^ Although perceived peer norms favorable to handgun carrying are identified as a risk factor for handgun carrying among adolescents,^20^ literature on norms among rural adolescents, especially qualitative data allowing for adolescent voices, is limited.

The present study addressed this gap by using a convergent mixed-methods design and community-based participatory research approach, merging qualitative and quantitative findings, to obtain a comprehensive understanding of the cultural context of firearms among rural adolescents. Among the benefits of using a mixed-methods design is the inclusion of individual lived experience, an element critical in intervention development for long-term feasibility and sustainability.^21^ In addition, community-based partnerships with adults have shown promise in developing and disseminating acceptable injury prevention messaging,^22^ and community partnerships with adolescents have been shown to support the development of youth-centered interventions for violence prevention.^23^ This mixed-methods study aimed to describe rural adolescents’ experiences with firearms and characterize firearm-related norms within their community, peers, and family.

## Methods

In this cross-sectional study, we administered a survey, followed by semistructured focus groups or interviews, between September 1, 2021, and September 30, 2022, to adolescents meeting the following inclusion criteria: residing in rural Washington state and enrolled in a county or tribal Washington State University (WSU) Extension 4-H youth development program as an intermediate or senior age-level grouping (ie, aged 12-19 years). The institutional review board at WSU reviewed and approved the present study. The Strengthening the Reporting of Observational Studies in Epidemiology (STROBE) reporting guideline for cross-sectional studies was followed as appropriate. Caregivers provided consent via an online or paper consent form. Adolescent assent was obtained prior to the first survey question and at the start of the focus group or interview. Assent procedures outlined confidentiality from parents or other adults to the extent allowable by law.

### Community-Based Participatory Research Approach

The project used a community-based participatory research approach by leveraging the WSU Extension System, a network of county- and tribal-based offices, personnel, and programs. eTable 1 in Supplement 1 outlines the core components of community-based participatory research and how they were applied in the present study.^24^ Extension personnel were engaged throughout the research process from planning to dissemination. Project year 1 (2020-2021) focused on planning and preparing for recruitment and year 2 (2021-2022) for data collection. In year 1, project staff and WSU Extension partners “laid the groundwork”^25^ by determining inclusion criteria, obtaining institutional review board and tribal research permit approval, and developing a recruitment plan.^25^ In year 2, the team initiated recruitment, revised recruitment to address emergent barriers, and collected participant data. Additional details are provided in the Consolidated Criteria for Reporting Qualitative Research (COREQ) reporting guideline checklist (eTable 2 in Supplement 1).^26,27^

### Data Collection

Recruitment was conducted in partnership with the WSU Extension 4-H youth development program. 4-H is one of the largest and oldest youth development organizations in the US and offers interest-based clubs, including shooting sports. We aimed to recruit half of the sample from 4-H shooting sports, an educational project using shooting sports as a vehicle for positive youth development. 4-H provided a roster of current and past enrolled adolescents, contact information (eg, zip code and guardian email and telephone number), and club enrollment. Information on race and ethnicity was collected for demographic comparison with overall 4-H enrollment (eTable 3 in Supplement 1).^28^ Participants self-reported race and ethnicity. Categories for racial and ethnic groups mirrored the Washington state youth surveillance survey, the Healthy Youth Survey.^29^ Recruitment was conducted via e-mail, telephone, and social media and by 4-H personnel.^30^ Adolescents were offered an in-person focus group, virtual focus group, or virtual interview to accommodate personal preference and schedule. Prior to a focus group or interview, adolescents completed the online survey. Focus groups and interviews were conducted in person and online to allow for flexibility in scheduling (see eTable 2 in Supplement 2 for data collection details).

### Measures

All measures were framed within social ecological levels of community, family, peer, and individual. In the survey, participants self-reported demographic characteristics (eg, gender identity, race and ethnicity, and zip code) and responded to items on neighborhood and personal safety, laws and norms favorable to carrying firearms, peer norms, family norms and monitoring, household firearm storage, individual history of firearm engagement, and firearm-related motives, access, and training. Items were drawn or adapted from existing surveys and measures.^31^ Qualitative questions sought to elicit perspectives at the intersection of constructs and levels (eg, family-level [level] norms [construct]). Survey items and questions used in the present study as well as contextual level and construct targets are shown in eTable 4 in Supplement 1.

### Statistical Analysis

Mixed-methods integration occurred within the study design, methods, and interpretation by matching constructs across quantitative survey items and qualitative prompts, using an identical sample for quantitative and qualitative data collection, and by weaving results or merging quantitative and qualitative findings and organizing them by broader themes to obtain meta-inferences in a joint display.^32^ See eTable 2 in Supplement 1 for a reporting organized by COREQ and additional detail.

Focus group and interview data were analyzed using a rapid qualitative analysis approach^33^ and conducted in 2 phases. After completing analyst training, the first phase included a standardized template to summarize responses from each transcript on a given construct using key words, preferably with participants own words (ie, in vivo). For the second phase, summary template data were merged into a matrix combining all transcripts by construct of interest. Using the matrix, the first author (E.H.W.) and 3 analysts separately identified similarities, differences, and trends in responses to generate broader themes. Discrepancies among analysts were discussed until there was unanimous agreement in themes. To ensure triangulation, the third author and community partner (A.H.) reviewed themes and suggested revised language and wording based on their expertise.

Survey data were analyzed using SPSS, version 26 for Windows (IBM Corp). Descriptive statistics were calculated to identify frequencies of responses among adolescents. Qualitative and quantitative data were compared and integrated using a weaving approach, in which findings were presented together and organized by theme.

## Results

The sample included 93 rural adolescents from across Washington state who were enrolled in 4-H (mean [SD] age on survey date, 15.7 [1.7] years; 49 female participants [52.7%] and 44 male participants [47.3%]; 7 American Indian or Alaska Native participants [7.5%], 2 Asian participants [2.2%], 4 Black participants [4.3%], 9 multiracial participants [9.7%], 3 Native Hawaiian or Other Pacific Islander participants [3.2%], and 86 White participants [92.5%]) (Table 1). All adolescents were confirmed to be living in rural settings, based on the National Center for Health Statistics definition of places smaller than “large central metro,”^34^ and 16 adolescents (17.2%) were classified as living in frontier and remote settings.^35^ Almost half the adolescents (44 [47.3%]) were enrolled in a 4-H shooting sports club.

**Table 1.** Sample Demographic Characteristics^a^

Demographic characteristic	No. (%) (N = 93)
Gender identity	
Female	49 (52.7)
Male	44 (47.3)
Race^b^	
American Indian or Alaska Native	7 (7.5)
Asian	2 (2.2)
Black	4 (4.3)
Multiracial	9 (9.7)
Native Hawaiian or Other Pacific Islander	3 (3.2)
White	86 (92.5)
Ethnicity	
Hispanic or Latino	6 (6.5)
Age, mean (SD), y	15.7 (1.7)
Age at survey date, y	
12	1 (0.8)
13	5 (4.5)
14	20 (19.2)
15	21 (21.6)
16	17 (18.6)
17	14 (16.3)
18	7 (8.6)
19	8 (10.4)
Rurality	
NCHS, mean (SD), No.	3.7 (1.4)
Large central metropolitan	0
Large fringe metropolitan	20 (21.5)
Medium metropolitan	34 (36.6)
Small metropolitan	8 (8.6)
Micropolitan	18 (19.4)
Noncore	13 (14.0)
Frontier and remote level 1	16 (17.2)
4-H youth development	
Shooting sports	44 (47.3)
Not in shooting sports	49 (52.6)

Abbreviation: NCHS, National Center for Health Statistics.

^a^ Sample demographic characteristics were self-reported and represent participants who completed the survey and participated in a focus group or interview.

^b^ Participants could select more than 1 response option. Those reporting more than 1 race self-reported as Black and White; Native Hawaiian or Other Pacific Islander and White; American Indian or Alaska Native and White; American Indian or Alaska Native and Asian and Native Hawaiian or Other Pacific Islander. Specific cell sizes are not reported for confidentiality reasons.

### Adolescent Handgun Behaviors

In this sample, 52 participants (55.9%) had carried a handgun at some point in their lives; among those, the mean (SD) age at first carry was 10.9 (3.1) years (Table 2). Across all adolescents, 21 (22.6%) had carried at least once in the past year, and 5 (5.4%) had carried at least once in the past month. A total of 4 adolescents (4.3%) reported carrying in the past year on school property, with 1 (1.1%) reporting carrying on school property in the past month.

**Table 2.** Individual Handgun Carrying Behaviors by Overall Sample and 4-H Shooting Sports

Behavior	No. (%)		
	Overall sample (N = 93)	Shooting sports (n = 44)	Not in shooting sports (n = 49)
Ever carried handgun	52 (55.9)	24 (54.5)	28 (57.1)
Age at first handgun carry, mean (SD), y	10.9 (3.1)	10.2 (3.1)	11.5 (3.1)
Past-year handgun carrying			
Never carried	41 (44.1)	20 (45.5)	21 (42.9)
Carried, but not in past year	31 (33.3)	15 (34.1)	16 (32.7)
1 or 2 Times	10 (10.8)	6 (13.6)	4 (8.2)
3-5 Times	6 (6.5)	1 (2.3)	5 (10.2)
6-9 Times	1 (1.1)	0	1 (2.0)
10-19 Times	1 (1.1)	1 (2.3)	0
20-29 Times	3 (3.2)	1 (2.3)	2 (2.0)
Past-month handgun carrying			
Never carried in lifetime	41 (44.1)	20 (45.5)	21 (42.9)
Carried, but not in past year	31 (33.3)	15 (34.1)	16 (32.7)
Carried, but not in past month	16 (17.2)	35 (79.5)	37 (75.5)
1 d	3 (3.2)	2 (4.5)	1 (2.0)
2-3 d	2 (2.2)	0	2 (4.1)
Past-year handgun carrying on school property			
Never carried	41 (44.1)	20 (45.5)	21 (42.9)
Carried, but not in past year	48 (51.6)	23 (52.3)	25 (51.0)
1 or 2 Times	3 (3.2)	1 (2.3)	2 (4.1)
6-9 Times	1 (1.1)	0	1 (2.0)
Past-month handgun carrying on school property			
Never carried in lifetime	41 (44.1)	20 (45.5)	21 (42.9)
Carried, but not in past year	48 (51.6)	23 (52.3)	25 (51.0)
Carried, but not in past month	3 (3.2)	0	3 (6.1)
1 d	1 (1.1)	1 (2.3)	0

### Firearm-Related Norms Themes

Three themes about rural adolescents’ firearm-related social norms emerged from the integrated qualitative and quantitative data: acceptable use, reason for carrying, and carrying in the community. Each theme also included subcategories. Table 3 illustrates a joint display of themes supported by qualitative quotes and quantitative data and resulting meta-inferences.

**Table 3.** Joint Display Presenting Integrated Themes, Qualitative Quotes, Quantitative Data, and Meta-Inferences

Themes and categories	Qualitative quotes	Summary and quantitative survey data	Meta-inference
**Theme 1: acceptable use**			
Legality: firearm-related laws and policies	“[It is appropriate to carry a handgun] anywhere that’s legal, like if you carrying is legal and your gun is legal. Just if it’s not against the law, I think it’s fine to carry.” “…if you’re going to carry a firearm for hunting the age isn’t as important as it is whether you have your [hunting] license or not…”	Over half of adolescents thought a peer would get caught by law enforcement if they carried a handgun. “If a kid carried a handgun in your neighborhood, would he or she be caught by law enforcement?” (N = 92) • Yes!: 18.3% (n = 17) • Yes: 38.7% (n = 36) • No: 34.4% (n = 32) • No!: 7.5% (n = 7)	Acceptable firearm behaviors reflected alignment with laws permitting minors to use a firearm.
Adult supervision: having an adult present when using a handgun	“You’ll need to be concerned [about safety] if [peers are] not carrying in a safe manner, or if they’re not carrying with their parents’ permission and if they are carrying it as like a joking thing.” “[My family feels that carrying] under supervision, it’s fine. If you’re not under… If you’re not under supervision, you might as well not be doing it because (a) it’s illegal and (b) you can get into serious trouble, or possibly hurt somebody else.”	Most adolescents reported they would be caught by parents if they carried a handgun without permission. “If you carried a handgun without your parent or guardian’s permission, would you be caught by your parent(s)/guardian(s)?” (N = 91) • Yes!: 53.8% (n = 50) • Yes: 26.9% (n = 25) • No: 9.7% (n = 9) • No!: 7.5% (n = 7)	There was general agreement that using a handgun required adult supervision for adolescents.
Training: firearm-related formal or informal instruction	“[It’s unacceptable for someone to carry if] they’re not trained and they’re going to be one of those people that like carry it not knowing how to use it.” “I think the key to [an adolescent carrying a firearm] being appropriate is as long as you’re well trained on it, like you guys [referring to other adolescents] do rifle here [at a 4-H shooting sports event]. I’m pretty confident you’re all trained. And so, I don’t see how that wouldn’t be appropriate. Sure. You’re underage, but you’re trained. You it’s, it would be more appropriate for you guys to do it than an adult that’s like 30, 40, but not trained at all.”	Most adolescents reported receiving some form of firearms training. “Has a family member, other adult, or organization trained you in safe use (eg, muzzle awareness, ensuring range is clear before firing)?” (N = 93) • Yes, I have received training for handguns and/or long guns: 81.7% (n = 76) • No, I did not receive training: 18.3% (n = 17)	There was general agreement on the importance of training prior to using or carrying a firearm.
**Theme 2: reason for carrying**			
Defense of self and/or property: using a handgun for protection of self or personal property	“There’s lots of farms out by where I live, so [people in my community carry firearms] for animals attacking other animals, for having to put down an animal that may be sick or broke its leg or something like that.” “My parents aren’t really in favor of people my age carrying handguns. They realize the kids are kids and we’re all kind of stupid sometimes. And if I was carrying a handgun for personal protection of that kind can lead to bad circumstances.”	Few adolescents carried a handgun for self-protection in the past month. “During the past 30 d, on how many days did you carry a handgun for self-protection? (Does not include carrying a handgun for hunting, fishing, or camping.)” (N = 93) • 0 d: 94.6% (n = 88) • 1 d: 3.2% (n = 3) • 2-3 d: 1.1% (n = 1) • 4-5 d: 1.1% (n = 1) • ≥6 d: 0.0% (n = 0)	It was acceptable among families and communities, but generally unacceptable for adolescents to carry a handgun for personal protection.
Recreation: using a handgun for enjoyment and/or subsistence	“I would say, one of the main reasons is for hunting, because hunting is a huge part of our culture, and that’s for some people. That’s how they like feed themselves during the winter.” “We just like we have a little mound of dirt and logs out back, and we have a couple of little steel plates out there, and we have like little rounds of things we see, and who can hit it the most times. It’s pretty fun.”	Most adolescents carried a handgun for transport to/from a shooting range. “When you carried a handgun, what was the primary reason you carried the gun?” (n = 49) • For transporting the gun to and from the shooting range: 67.3% (n = 33) • Protection against animals: 16.3% (n = 8) • Protection against strangers: 6.1% (n = 3) • For transporting the gun to and from work: 2.0% (n = 1) • For protection against people I know: 0.0% (n = 0) • For some other reason: 8.2% (n = 4)	Firearms as tools for enjoyment, skill building, and subsistence was acceptable.
Reasons other than self-defense or recreation: using a handgun for purposes other than self-defense or recreation	“For the most part unless it’s for sport, but no one really just carries it to carry it, they have a reason.” “Like just carrying it for no reason. They [my family] probably wouldn’t whatever the word is, but like having it for competitions or, and you have all the training and the skills and you have it for like a club or 4-H then they’d be like comfortable with it and stuff. But just carrying it for no reason, not so much.”	Most adolescents reported people residing with them carried a handgun for reasons other than work or hunting in the last year. “How many times in the past year (12 mo), did people who live in the same residence as you carry a handgun (other than while hunting, shooting targets, or as part of your job)?” (n = 54) • Never: 13.0% (n = 7) • 1 or 2 times: 16.7% (n = 9) • 3-5 times: 20.4% (n = 11) • 6-9 times: 11.1% (n = 6) • 10-19 times: 9.3% (n = 5) • 20-29 times: 9.3% (n = 5) • 30-39 times: 1.9% (n = 1) • ≥40 times: 18.5% (n = 10)	Adolescents generally agreed that firearms for purposes outside of recreation and self-defense were unacceptable for them, but it was not uncommon for adults to do so.
To cause harm or intimidate: using a handgun to cause physical or psychological harm to others	“[Handguns are] not just for killing people. Because that’s what typically is seen in social media is that they kill people. But it’s not just about that.” “Kind of, like she [other participant] said, like being judged about carrying, seeing that you just have it because you want to feel [you]… have power over other people or you think the people around you are dangerous, so that’s why you carry the gun and stuff.” “…because some people for them it’s the pride they want to be seen and scare us. They want to infringe a sense of fear to others, so that you can just keep off from them.”	Most adolescents reported no or little chance they would be seen as “cool” if they carried a handgun. “What are the chances you would be seen as cool if you carried a handgun?” (N = 93) • No or very little chance: 61.3% (n = 57) • Little chance: 20.4% (n = 19) • Some chance: 12.9% (n = 12) • Pretty good chance: 4.3% (n = 4) • Very good chance: 1.1% (n = 1)	It was unacceptable to carry firearms with the intention of showing off or harming another person, even though such behaviors had occurred in their communities.
**Theme 3: carrying in the community**			
Carrying in public spaces: carrying within community and public spaces	“I’d say [it is not appropriate to carry] in places like [other participant] said, like schools, because, hopefully, there will not be anyone there that will be a threat and also hospitals, because it is just dangerous, there’s a lot going on at hospitals, and anything could happen, shotgun can accidentally be fired.” “In my community, I think people mostly carry like a handgun, because I’ve seen in my school the kids that carry handguns like right here [gestures to belt area].”	Most adolescents reported carrying a firearm to school was wrong. “How wrong do you think it is for someone your age to take a handgun to school?” (N = 93) • Not wrong at all: 0.0% (n = 0) • A little bit wrong: 6.5 (n = 6) • Wrong: 10.8% (n = 10) • Very wrong: 82.8% (n = 77)	It was unacceptable for adolescents to carry a handgun in public spaces even though such behaviors occurred in their communities.
Inconsistent community experiences: wide range of responses to community handgun behavior	“I don’t see a lot of open carrying in my community. Probably a lot of concealed carry. I just don’t see a lot of it.” Participant 1: “Wait let me think about it, I don’t, I don’t know if there’s ever not be an appropriate time [to carry a firearm], I mean. Probably if it’s not like… I don’t know, I don’t know.” Participant 2: “I agree with [Participant 1] on that.” Participant 3: “I live in a super religious suburb where there are pretty much no guns at all. I don’t think any of our neighbors hold any.” Participant 4: “Well, I live in the same place you [Participant 3] do. I think I disagree with that a little bit. I think a lot of people have guns there. Yeah.”	Only half of adolescents reported it would be very hard to access a handgun from a community member. “If you wanted to get a handgun, how easy would it be for you to borrow one from someone in your community?” (N = 93) • Very easy: 5.4% (n = 5) • Sort of easy: 12.9% (n = 12) • Sort of hard: 31.2% (n = 29) • Very hard: 50.5% (n = 47)	There was variation in perceived frequency, type, and availability of handguns within their communities.

#### Acceptable Use

Theme 1 centered on acceptable ways to engage with a firearm, which aligned with laws permitting firearm use among minors and had 3 subcategories: (1) legality, (2) adult supervision, and (3) training. Legality captured how descriptions of adolescents’ acceptable firearm use aligned with related laws and policies. Even when adolescents did not explicitly mention state laws outlining legal exemptions for minors to use a firearm, they articulated awareness of such factors (eg, permit requirements) and referenced what was or was not legal. In describing individual, peer, and family behavior, 75 adolescents (80.6%) indicated that using a firearm without adult supervision was unacceptable and that they would be caught by parents if carrying a handgun without permission. Although this finding aligns with firearm-related laws and policies, it was mentioned consistently enough to emerge as a separate category. Adolescents emphasized the importance of community members, peers, and family members receiving training prior to using a firearm, and most adolescents (76 [81.7%]) had received some form of formal or informal firearm use training.

#### Reason for Carrying

Theme 2 contained 4 subcategories associated with the reason for carrying a firearm. Two were acceptable: (1) defense of self and/or property and (2) recreation, whereas 2 were unacceptable: (3) reasons other than self-defense or recreation and (4) to cause harm or intimidate. Adolescents generally felt it was acceptable in their communities and families for adults to carry for the protection of one’s person, family, and/or property, but it was unacceptable for adolescents to do so. Nevertheless, 5 adolescents (5.4%) reported doing so in the past month. Using a firearm for recreation (eg, hunting and shooting sports) was common (33 of 49 adolescents [67.3%] last carried a handgun to or from the shooting range) and acceptable among adolescents, their families, and communities, as was using firearms as a tool for enjoyment, skill building, subsistence, and cultural connection. Adolescents agreed that carrying for no reason at all or to cause psychological harm (eg, intimidation) or physical harm (eg, injury) was unacceptable for them but occurred within their community and sometimes among their peer group. Similarly, most adolescents (76 [81.7%]) reported they would not be seen as “cool” (ie, injunctive norm) if they carried a handgun.

#### Carrying in the Community

Theme 3 related to firearm-carrying behaviors in the community and included 2 subcategories of (1) carrying in public spaces and (2) inconsistent community experiences. There was generally agreement that it was unacceptable for adolescents to carry a handgun in public spaces where people gather (eg, churches, grocery stores, and schools). This finding was reflected in survey data in which participants indicated that bringing a handgun to school was very wrong or wrong (87 [93.5%]). Perceptions of carrying frequency within communities were inconsistent, ranging from never to always. Across adolescents, there was variation in the type of carrying perceived to occur in the community (eg, concealed carry), as well as the presence of handguns within their communities. This variation occurred even among adolescents living in the same community. For example, 47 adolescents (50.5%) reported it would be very hard to borrow a handgun from someone in the community.

## Discussion

In this cross-sectional study of adolescents in rural Washington state, findings centered around firearm-related community, family, and peer social norms (eg, acceptability). The use of a mixed-methods design allowed for amplification of adolescent voices, highlighted by the following candid participant quote: “Well, nobody asks us these questions. So being able to actually outright talk about it [firearms] with other than family, it’s good to be able to voice our opinions and somewhat be heard by people.” This study contributes to the limited but growing research on rural adolescents’ experiences with firearms.

Adolescents in the present study initiated carrying firearms at a mean (SD) age of 10.9 (3.1) years. This age is younger than in studies of urban adolescents.^36^ Adolescents in the present study reported higher rates of past-year handgun carrying (21 [22.6%] carried once or more in the past year) than nationally representative samples of rural adolescents (range, 6.3%-12.4%) and urban adolescents (range, 3.6%-5.0%).^3^ Rates of handgun carrying on school property were similar to those found in other rural samples (4 adolescents [4.3%] in the present study vs 3%).^37^ Initiation age and recent carrying behavior are associated with reasons for carrying. Prior research on handgun carrying among urban adolescents found that most reported carrying a handgun for self-defense or protection, to confer respect (eg, flashing a firearm), or to fit in and that carrying was associated with antisocial behaviors.^3,20^ In comparison, rural adolescents in the current sample predominantly carried firearms for reasons viewed as prosocial in rural settings (recreation, hunting, and sport) and identified that it was unacceptable for adolescents to carry for protection, self-defense, or intimidation (eg, showing off). These findings may reflect contextual differences in urban and rural areas. For example, due to historical and ongoing systems of oppression, rates of firearm homicide and other violent crime are concentrated in urban, primarily low-income, areas.^38^ The concentration of interpersonal violence, in turn, is associated with young people carrying guns for fear of personal safety.^20^

Social norms have been implicated in individual health behaviors and are often a focus of harm reduction and health promotion approaches supporting individual behavior change.^39^ What was acceptable or unacceptable was generally consistent among adolescents and their peers, although adolescents acknowledged some peers engaged in unacceptable behaviors anyway. The legality subcategory represented the broadest or most inclusive threshold for whether a behavior was acceptable or unacceptable and aligned with state law permitting minors to carry a firearm. This supports findings that stronger state-level firearm laws are associated with less adolescent handgun carrying.^40^ Discrepant social norms tended to be associated with age—what was acceptable for adults, such as carrying firearms at all times, was not always acceptable for adolescents. Findings aligned with a recreational, rather than self-defense, firearm subculture.^5^ It was normative and acceptable for adolescents and adults around them to carry handguns for recreation and for adults to carry firearms for protection of person and personal property from wild animals and not crime.^41^

Findings hold implications for firearm injury prevention efforts in rural communities, especially around enhancing training and developing norms-based approaches. Adolescents emphasized training as important prior to using a firearm, supporting prior research pointing to safety education as a key component in firearm injury prevention efforts.^42^ Approaching injury prevention from a safety perspective was instrumental for participant recruitment in the present study^28^ and is generally supported by rural communities.^42,43^ This means that enhancing safety training may be an acceptable option to, for example, encourage less lethal tools (eg, bear spray) as a harm reduction measure. Social norms are especially influential on adolescent behavior and can be used in injury prevention efforts to validate normative behavior and correct misperceptions. Similar normative approaches have demonstrated successful effects on adolescent alcohol use.^44^ Validating normative behavior and correcting misperceptions about firearms are important, as rural adolescents are at increased risk of firearm-involved suicide,^38^ and future research is needed to better understand the intersection of mental health–related and firearm-related social norms to inform culturally acceptable suicide prevention efforts at community, family, and peer levels. Social stigma surrounding pursuing mental health treatment and social acceptability around firearm use and changes in storage practices in response to mental health concerns may be important place-based risk factors for rural adolescents. Suicide and mental health were not measured or discussed in focus groups, but rural adolescent perspectives on firearm access or firearm-related behaviors, such as handgun carrying during a mental health crisis, is an important area for future research.

### Limitations

This study has some limitations. Social desirability bias may have affected adolescents’ responses and engagement. It is plausible that typically socially unacceptable behaviors (eg, carrying a firearm to show off) were underreported in this study. We acknowledge that generalizability is not the goal of qualitative or mixed-methods research. Rather, the goal of the present study was to provide rich description to expand our understanding of rural adolescents’ experiences with firearms. Our sample provides the perspectives of a specific group of adolescents living within rural Washington state participating in 4-H youth development, including multiple genders, races and ethnicities, and, importantly, voices of tribal-enrolled adolescents. These experiences or perspectives may not apply to other rural populations or settings.

## Conclusions

Rural adolescent handgun carrying is increasing, resulting in greater risk for firearm-involved injury and death.^45^ To reduce firearm-involved deaths among rural adolescents, we need to better understand how they perceive and engage with firearms. This cross-sectional study demonstrated that rural adolescents desire to be engaged on this topic, and future youth participatory research is crucial for developing culturally and developmentally relevant firearm injury prevention programs. Integrated qualitative and quantitative findings from the present study suggest that rural adolescents generally differentiated between acceptable and unacceptable handgun carrying behaviors and point to key contextual differences for rural adolescent behaviors, such as carrying a handgun predominantly for hunting, recreation, or sport, and adult behavior, such as carrying for protection of person and personal property. Understanding firearm-related social norms in rural communities holds implications for culturally appropriate firearm injury prevention efforts, especially associated with enhancing training, developing norms-based prevention approaches, and tailoring efforts to rural settings.

## Notes

Supplement 1. **eTable 1.** Core Components of Community-Based Participatory Research (CBPR) and Application in the Current Study **eTable 2.** Completed COREQ Checklist **eTable 3.** Demographics of Sample and All Enrolled 4-H Members **eTable 4.** Qualitative and Quantitative Items Used in the Current Study Organized By Socio-Ecological Context and Construct

Supplement 2.
Data Sharing Statement
