# Supplementary material for: Firearm Experiences, Behaviors, and Norms Among Rural Adolescents
Source: JAMA Netw Open. 2024 Oct 24;7(10):e2441203. doi: 10.1001/jamanetworkopen.2024.41203 (PMC11581587; doi:10.1001/jamanetworkopen.2024.41203)
Supplement: Supplement 1. — eTable 1. Core Components of Community-Based Participatory Research (CBPR) and Application in the Current Study eTable 2. Completed COREQ Checklist eTable 3. Demographics of Sample and All Enrolled 4-H Members eTable 4. Qualitative and Quantitative Items Used in the Current Study Organized By Socio-Ecological Context and Construct [file jamanetwopen-e2441203-s001.pdf]

Supplementary Online Content

Weybright EH, Terral HF, Hall A, et al. Firearm experiences, behaviors, and norms among rural adolescents. *JAMA Netw Open.* 2024;7(10):e2441203. doi:10.1001/jamanetworkopen.2024.41203

eTable 1. Core Components of Community-Based Participatory Research (CBPR) and Application in the Current Study

eTable 2. Completed COREQ Checklist

eTable 3. Demographics of Sample and All Enrolled 4-H Members

eTable 4. Qualitative and Quantitative Items Used in the Current Study Organized By Socio-Ecological Context and Construct

This supplementary material has been provided by the authors to give readers additional information about their work.

eTable 1. Core Components of Community-Based Participatory Research (CBPR) and Application in the Current Study

Component	Description[24]	Application in Current Study
Forming a CPBR relationship.	“This involves such processes as identifying potential partners and communities to be involved, building trust and relationships, establishing operating norms and CBPR principles to ensure equity and power sharing, and creating an infrastructure for carrying out the research process.” (p.12)	Leveraged an ongoing (3+ years) partner relationship between the first author in a campus-based department and third and fourth authors in Extension 4-H Youth development, specifically engaging the 4-H Shooting Sports program coordinator and advisory committee members.
Assessing community strengths and dynamics.	“This involves asking questions, such as: What are the strengths and resources in the community? What are key cultural and historical dimensions? Which are the influential organizations? Where’s the power in the community? Who needs to be involved to ensure community voice?” (p.11)	Group discussion with Extension partners on youth firearm-related issues, opportunities, and activities within the community. Reached out to additional Extension personnel as well as 4-H youth and caregivers.
Identifying priority health concerns and research questions.	“Key questions here include: What are the major health problems that have an impact on the community that a partnership might address? How are these problems identified and prioritized? What are the factors (for example, social, economic) that contribute to these health concerns? What are the key research questions that this study is intended to answer?” (pp. 11-12)	With Extension partners, discussed key factors related to youth development and firearms in rural communities to inform research questions and design. Aligned discussion with empirical literature on risk and protective factors for firearm-related injury.
Designing and conducting research.	“This involves, for example, deciding which research design and data collection methods to use, and, as appropriate, what the most appropriate intervention strategy is, as well as determining how to implement the design and the strategies selected.” (p.12)	Engaged Extension partner group in discussion for initial planning for funding proposal. Once funded, engaged group in discussion in Year 1 planning year and throughout Year 2 data collection. Revisions were ongoing based on partner feedback. 4-H youth and caregivers were engaged in recruitment and data collection, and engagement opportunities were aligned with existing community projects and events.
Feeding back and interpreting research findings.	“This involves sharing the findings from the research, such as the results of the analysis of survey or in-depth interview data, and engaging all partners in making sense of what was found.” (p.12)	Extension partners participated in analyses, discussion, and drafting study findings and related interpretations. 4-H youth and caregivers were engaged in interpretation and triangulation of findings.
Disseminating and translating research findings.	“Critical questions here include: What is most important from the findings to share with the community? What are the most appropriate ways to disseminate results to the community? What is the role of community partners in publishing the results? How can the results be translated	Extension partners included as co-authors on scholarly publications and presentations. Engaged partners in disseminating findings to 4-H Youth Development personnel, volunteers, youth, and caregivers.

© 2024 Weybright EH et al. *JAMA Network Open.*

	and disseminated into more broadscale interventions and policy change?” (p.12)	
Maintaining, sustaining, and evaluating the partnership.	“An ongoing process that is at the center of all these phases and occurring throughout them. Relevant questions to address include: How well is the partnership working? How can the partnership process be improved? What aspects of the partnership need to be considered regarding sustainability, for example, enhanced relationships and capacity?” (p.12)	Held monthly or bi-monthly meetings with Extension partners to check in on project activities, discuss challenges, and collectively problem solve.

© 2024 Weybright EH et al. *JAMA Network Open.*

eTable 2. Completed COREQ Checklist

Item	Guide Questions	Application in Current Study
1. Interviewer /facilitator	Which author/s conducted the interview or focus group?	Focus groups and interviews were led by a facilitator and co-facilitator. Facilitators included co-authors (AH, GV) and graduate research assistants (ED, EP). Co-facilitators included graduate research assistants (ED, EP) and Extension staff (ML, KJ, JF, LM, JS).
2. Credentials	What were the researcher's credentials?	Facilitator credentials included doctoral degree (AH, GV), Master’s degree (ED), and graduate student (EP).
3. Occupation	What was their occupation at the time of the study?	Facilitators were employed as Extension faculty (AH, GV) and graduate students (ED, EP).
4. Gender	Was the researcher male or female?	All facilitators and co-facilitators identified as female except for GV who identified as male.
5. Experience and training	What experience or training did the researcher have?	All facilitators had conducted qualitative focus groups or interviews prior to serving in the role and were trained in the project data collection protocol.
6. Relationship established	Was a relationship established prior to study?	Yes, Extension facilitators had pre-existing relationship with participants as 4-H enrolled youth.
7. Participant knowledge of the interviewer	What did the participants know about the researcher?	Facilitators used a focus group or interview protocol with a script which stated: “We [facilitators] are here at [county/city name] trying to learn about your opinions and experiences related to firearms.” Participants knew of the facilitators, where they worked, and purpose of research.
8. Interviewer characteristics	What characteristics were reported about the facilitator?	Facilitators self-disclosed personal interest in research topic and desire to hear from participants.
9. Methodological orientation and Theory	What methodological orientation was stated to underpin the study?	Study was underpinned by Social Ecological Model and Theory of Planned Behavior. Analysis was based on rapid qualitative analysis.
10. Sampling	How were participants selected?	A purposive sample of 4-H enrolled youth was recruited. Researchers obtained a roster of 4-H enrolled youth. We purposively recruited half of the sample from 4-H Shooting Sports enrolled youth.
11. Method of approach	How were participants approached?	Participants were approached via email, phone, 4-H social media, and word of mouth from local 4-H personnel and volunteers.
12. Sample size	How many participants were in the study?	*N*=93.
13. Non-participation	How many people refused to participate or dropped out? Reasons?	One participant completed the survey but did not participate in a focus group or interview due to scheduling conflict.
14. Setting of data collection	Where was the data collected?	We conducted 39 focus groups and individual interviews (18 focus groups, 21 interviews). Of the focus groups, 11 were virtual (i.e., Zoom), six in-person, and one was hybrid. All 21 interviews were virtual. Of the in-person focus groups, 3 took place on-site at scheduled 4-H events (Shooting Sports, horse camp), one on a tribal

© 2024 Weybright EH et al. *JAMA Network Open.*

		reservation, and the remainder at county Extension offices. The survey was collected online via Qualtrics.
15. Presence of non-participants	Was anyone else present besides the participants and researchers?	The focus group or interview only included participants, facilitator(s), and co-facilitator(s).
16. Description of sample	What are the important characteristics of the sample?	The sample was comprised of rural adolescents, which is the key characteristic.
17. Interview guide	Were questions, prompts, guides provided by the authors? Was it pilot tested?	The focus group and interview protocol (including topics and prompts) were initially generated by the research team and piloted with a group of 4-H youth.
18. Repeat interviews	Were repeat interviews carried out?	No repeat interviews were carried out.
19. Audio/visual recording	Did the research use audio or visual recording to collect the data?	In-person data were captured with a digital recorder. Virtual data were captured with Zoom audio recording. For both formats a transcript was generated and checked for accuracy.
20. Field notes	Were field notes made during and/or after the interview or focus group?	Facilitators had the option of taking notes before, during, and after data collection. Notes were taken on the context of in-person events such as the location, site, etc. Facilitators and co-facilitators met to debrief after focus groups and interviews.
21. Duration	What was the duration of the interviews or focus group?	Focus groups lasted 45-60 minutes while interviews averaged 22 minutes. The survey lasted, on average, 12 minutes.
22. Data saturation	Was data saturation discussed?	Data saturation was discussed during data collection. Facilitators and other project staff met to discuss emergent and recurring topics and whether new topics were being introduced or not. Data saturation was confirmed prior to ending data collection.
23. Transcripts returned	Were transcripts returned to participants?	No, transcripts were not returned for comment or correction.
24. Number of data coders	How many data coders coded the data?	Initial coder training included the first author and three project staff independently coding the same transcript and then meeting to discuss instances of disagreement. This process was repeated to ensure consistency. Once initial coder training was complete, one coder coded each transcript and a second coder audited 20% (n=8) of all transcripts for accuracy and alignment with initial coding training.
25. Description of the coding tree	Did authors provide a description of the coding tree?	Themes and categories were provided in Table 3.
26. Derivation of themes	Were themes identified in advance or derived from the data?	Themes were derived from the data.

© 2024 Weybright EH et al. *JAMA Network Open.*

27.	Software	What software, if applicable, was used to manage the data?	Excel and word were used to manage the data and coding process.
28.	Participant checking	Did participants provide feedback on the findings?	No
29.	Quotations presented	Were participant quotations presented to illustrate the themes / findings? Was each quotation identified?	Quotations are included in the joint display (Table 3).
30.	Data and findings consistent	Was there consistency between the data presented and the findings?	Yes, data were triangulated to confirm emerging findings.[43] This included investigator triangulation (i.e., multiple individuals collecting and analyzing data), use of multiple methods (i.e., qualitative and quantitative data), and analyst triangulation (i.e., consistency and agreement across independent coders).
31.	Clarity of major themes	Were major themes clearly presented in the findings?	Major themes are labeled as such in the results and joint display (Table 3).
32.	Clarity of minor themes	Is there a description of diverse cases or discussion of minor themes?	Minor themes are referred to as categories in the results and joint display (Table 3).

Note: COREQ = Consolidated Criteria for Reporting Qualitative Research [44]

© 2024 Weybright EH et al. *JAMA Network Open.*

eTable 3. Demographics of Sample and All Enrolled 4-H Members

**Demographics**	**No. (%) or (*M*, SD)**	
	**Study Participants** *N*=93	**2020-2021 WA 4-H Enrollees** *N*=5247
Gender Identity		
Female	49 (52.7%)	3380 (64.4%)
Male	44 (47.3%)	1852 (35.3%)
Nonbinary	--	5 (0.1%)
Not provided/Prefer not to respond	--	10 (0.2%)
Race		
American Indian/Alaska Native	7 (7.5%)	191 (3.6%)
Asian	2 (2.2%)	101 (1.9%)
Black	4 (4.3%)	91 (1.7%)
Native Hawaiian or Pacific Islander	3 (3.2%)	23 (0.4%)
White	86 (92.5%)	4503 (85.8%)
More than one Race	9 (9.7%)	29 (0.6%)
Not provided/Prefer not to respond	--	309 (5.9%)
Ethnicity		
Hispanic or Latino	6 (6.5%)	278 (5.3%)
Age on Survey Date/Enrollment	(15.7, 1.7)	(13.4, 2.7)
12 years old	1 (0.8%)	926 (17.6%)
13 years old	5 (4.5%)	892 (17.0%)
14 years old	20 (19.2%)	912 (17.4%)
15 years old	21 (21.6%)	731 (13.9%)
16 years old	17 (18.6%)	631 (12.0%)
17 years old	14 (16.3%)	548 (10.4%)
18 years old	7 (8.6%)	438 (8.3%)
19 years old	8 (10.4%)	169 (3.2%)

Note. 4-H Enrollees taken from 2020-2021. 4-H Enrollment process limited participants to one race response. 4-H enrollees reported are restricted to those 12 to 19 years old to match study Inclusion criteria. Comparable rurality data are not available for 4-H enrollees due to zip code data not being available.

© 2024 Weybright EH et al. *JAMA Network Open.*

eTable 4. Qualitative and Quantitative Items Used in the Current Study Organized By Socio-Ecological Context and Construct

Construct	Qualitative Question and Probe	Quantitative Item and Response Options
Community Level		
Norms - Injunctive	When do you think it is appropriate to carry a firearm in your community?<br>• When is it not appropriate?<br><br>What are some of the reasons people choose to carry firearms in your community?<br>• What are some of the reasons that people choose NOT to carry firearms in your community?	If you wanted to get a handgun without purchasing, how easy would it be for you to borrow one from someone in your community?<br>• Very hard; Sort of hard; Sort of easy; Very easy<br><br>If a kid carried a handgun in your neighborhood, would he or she be caught by law enforcement?<br>• NO!; No; Yes; YES!
Norms - Descriptive	What does firearm carrying look like in your community?<br>• What types of firearms do people carry in your community?	
Behaviors		During the past 30 days, on how many days did you carry a handgun for self-protection? (DOES NOT include carrying a handgun for hunting, fishing, or camping.)<br>• 0 days; 1 day; 2-3 days; 4-5 days; 6 or more days
Peer Level		
Norms - Injunctive	How do people your age decide when to carry and when to not carry a firearm?<br>• What are some reasons why people your age choose to carry firearms?<br>• What are some reasons that people your age choose NOT to carry firearms?<br>When do you think it is appropriate for someone your age to carry a firearm?<br>• When is it not appropriate?	How wrong do you think it is for someone your age to take a handgun to school?<br>• Very wrong; Wrong; A little bit wrong; Not wrong at all<br><br>What are the chances you would be seen as cool if you carried a handgun?<br>• No or very little chance; Little chance; Some chance; Pretty good chance; Very good chance
Norms - Descriptive	What does firearm carrying look like among people your age?<br>• What types of firearms do people your age carry?<br>• How do people your age get access to firearms?	
Family Level		
Norms - Injunctive	How does your family feel about people your age carrying firearms?<br>• What, if any, safety concerns does your family have about people your age carrying firearms?	If you carried a handgun without your parent or guardian's permission, would you be caught by your parent(s)/guardian(s)?<br>• NO!; No; Yes; YES!
Norms - Descriptive	What does firearm carrying look like in your family?	How many times in the past year (12 months), did people who live in the same residence as you carry a handgun (other

© 2024 Weybright EH et al. *JAMA Network Open.*

	• What types of firearms do people in your family carry?	than while hunting, shooting targets, or as part of your job)?<br>• Never; 1-2 times; 3-5 times; 6-9 times; 10-19 times; 20-29 times; 30-39 times; 40 or more times
Behaviors	What activities does your family engage in related to firearms?	
Individual Level		
Behaviors		Have you ever carried a handgun?<br>• Yes; No<br><br>How old were you when you first carried a handgun?<br>• [write in age]<br><br>How many times in the past year (12 months), have you carried a handgun (other than while hunting, shooting targets, or as part of your job)?<br>• Never; 1 or 2 times; 3 to 5 times; 6 to 9 times; 10-19 times; 20-29 times; 30-39 times; 40 or more times<br><br>During the past 30 days, on how many days did you carry a handgun (other than while hunting, shooting targets, or as part of your job)?<br>• 0 days; 1 day; 2-3 days; 4-5 days; 6 or more days<br><br>How many times in the past year (12 months), have you taken a handgun to school?<br>• Never; 1 or 2 times; 3 to 5 times; 6 to 9 times; 10-19 times; 20-29 times; 30-39 times; 40 or more times<br><br>During the past 30 days, on how many days did you carry a handgun on school property?<br>• 0 days; 1 day; 2-3 days; 4-5 days; 6 or more days
Training		Has a family member, other adult, or organization trained you in the following... Safe use (e.g., muzzle awareness, ensuring range is clear before firing)<br>• No, I did not receive training; Yes, I received training for handguns; Yes, I received training for long guns; Yes, I received training for handguns and long guns

© 2024 Weybright EH et al. *JAMA Network Open.*

Motivation		When you carried a handgun, what was the primary reason you carried the gun?<br>• For protection against strangers; For protection against people I know; For protection against animals; For transporting the gun to and from work; For transporting the gun to and from the shooting range; For some other reason
