# Supplementary material for: Firearm Experiences, Behaviors, and Norms Among Rural Adolescents
Source: JAMA Netw Open. 2024 Oct 24;7(10):e2441203. doi: 10.1001/jamanetworkopen.2024.41203 (PMC11581587; doi:10.1001/jamanetworkopen.2024.41203)
Supplement: Supplement 2. — Data Sharing Statement [file jamanetwopen-e2441203-s002.pdf]

Data Sharing Statement

Weybright. Firearm Experiences, Behaviors, and Norms Among Rural Adolescents. *JAMA Netw Open*. Published October 24, 2024. doi:10.1001/jamanetworkopen.2024.41203

Data
Data available: No

Additional Information
Explanation for why data not available: Some data were obtained under a tribal research permit and therefore are owned by the respective tribe.
